# FT4 and TSH, relation to diagnoses in an unselected psychiatric acute-ward population, and change during acute psychiatric admission

**DOI:** 10.1186/s12888-018-1819-3

**Published:** 2018-07-28

**Authors:** Yuki Sakai, Valentina Iversen, Solveig Klæbo Reitan

**Affiliations:** 1 Faculty of Medicine and Health sciences, Institute for Mental Health, Norwegian University of Science and Technology, Trondheim, Norway; 2 Department of Mental health, St. Olav’s University Hospital, Trondheim, Norway

**Keywords:** Thyroid, TSH, FT4, Psychiatric, Stress, Acute, The HPT axis

## Abstract

**Background:**

Alteration in thyroid activity is a well-known cause of symptoms mimicking psychiatric disorders. There are reports on altered levels of thyroid hormones in patients with certain psychiatric disorders compared to healthy controls; still, the magnitude and importance of the phenomenon is not known. We wanted to explore the level of thyroid hormones in different diagnostic groups in an acute-psychiatric population. We also wanted to follow any change during their stay.

**Methods:**

Patients aged 18 years and older admitted to a closed, psychiatric inpatient ward were eligible if giving informed consent. For 539 patients representing all main psychiatric diagnostic groups and with equal gender distribution, data for FT4 were available for 539 patients, and data for TSH were available from 538 patients at admittance. For 239 patients, data for FT4 were available at both admittance and discharge, and the corresponding number for TSH was 236 patients.

**Results:**

A significantly higher share of patients had higher levels of FT4 and TSH at admittance than expected for healthy individuals. No significant effect of gender or most diagnostic groups was seen.

For female patients with substance-use disorder (SUD), the level of TSH was significantly lower than that for all other diagnostic groups. No other difference in the levels of FT4 and TSH was seen between the main diagnostic groups, and the effect in SUD was not seen in males.

For the population with available markers at both admittance and discharge, in total, there was a significant reduction of FT4 from admittance to discharge, not followed by any change in TSH.

**Conclusions:**

In acutely admitted psychiatric patients there seems to be an increased FT4 and TSH. FT4 is normalized during the inpatient stay independently of TSH. This indicates somatic effects of psychiatric stress that may be of clinical importance and the phenomenon should be further explored. Mainly different diagnostic groups did not differ in level of FT4 and TSH. Thus future studies on thyroid activity in psychiatric patients should focus on function and level of stress and suffering rather than diagnostic groups.

**Electronic supplementary material:**

The online version of this article (10.1186/s12888-018-1819-3) contains supplementary material, which is available to authorized users.

## Background

Clinical thyroidal disorders mimicking depression and anxiety are well known. Further, thyroid hormones (T3/T4) and thyroid-stimulating hormone (TSH) have been suggested to play a critical role in the regulation of neuronal growth and development, as well as in the alteration of neurocognitive functions including mood and cognition in the mature brain [1]. Although it is established that hypothalamic-pituitary-thyroid (HPT) axis abnormalities may be associated with psychiatric pathophysiology [2–4], the HPT pattern in psychiatric patients with non-thyroidal illness is still inconsistent. Altered concentrations of free thyroxine (FT4) have been described in patients with psychiatric disorders such as schizophrenia and affective disorders [3, 5–8], but findings are inconsistent; for example, other reports on personality disorder and alcoholism are negative [6]. In addition, an association between thyroid hormones within reference range and cognitive dysfunction in older people has been indicated [1]. The levels of TSH as well as TSH responses to thyrotropin - releasing hormone (TRH) have been described to be different in healthy controls compared to patients with psychiatric disorders, including major depression, bipolar mania, alcoholism, and borderline personality disorder [9, 10]. Thyroid disorders have been described as a risk factor for treatment resistant depression [11]. However, other studies do not find an association between the levels of thyroidal components and neuropsychiatric disorders [1, 12]. Dickerman and Barnhill [13] hypothesized that these considerable variations in thyroid alterations in psychiatric patients may be caused by a response to an underlying systemic illness or by the acute psychiatric illness itself. In line with this, altered levels of FT4 from acute state to remission have been described in schizophrenic patients in one study and attributed to medications [5], although it is far from being clear. To our knowledge, there are no studies on levels of thyroid hormones in a general psychiatric population at acute admittance.

To summarize, the connection between thyroid status and different psychiatric disorders is not consistently clear, and although an important effect of thyroid hormones on brain function is suggested, it is not clear whether acute psychiatric suffering has an effect.

Thus, the aim of this study was to investigate the relation between the levels of thyroid hormones and different psychiatric diagnoses in a general psychiatric acute inpatient population. In addition, we wanted to study the variation in thyroid status over an acute admission.

## Methods

### Aim

The first aim was to explore the prevalence of deviating thyroid levels in an unselected psychiatric acute-ward population and potential relations to diagnoses.

The second aim was to study whether there was a change in thyroid levels from admittance to discharge.

### Ethics

The study was part of a large study approved by the Norwegian Regional Committee for Ethics (REC, identification number 199/04) and by Middle Norway and Norwegian Social Science Data Services (NSD, identification number 11771).

### Design and setting of the study

The study was a cross-sectional study on the prevalence of thyroid levels and a follow up for change in thyroid levels in some of the participants.

Participants were recruited among patients admitted to the only closed, inpatient acute psychiatric ward for a population of 150.000 above the age of 18 years in South-Trøndelag county in the Middle of Norway in the period from 19.10.2004 to 30.11.2006. Clinical data and laboratory data according to standard clinical procedures in the hospital were available in the patients’ files. Patients were given information and asked to consent to participation in a study collecting data on several fields including thyroid status. For patients giving informed consent, the data were collected in a research database, and participants were made anonymous.

### Patients and patient characteristics

**Fig. 1:**
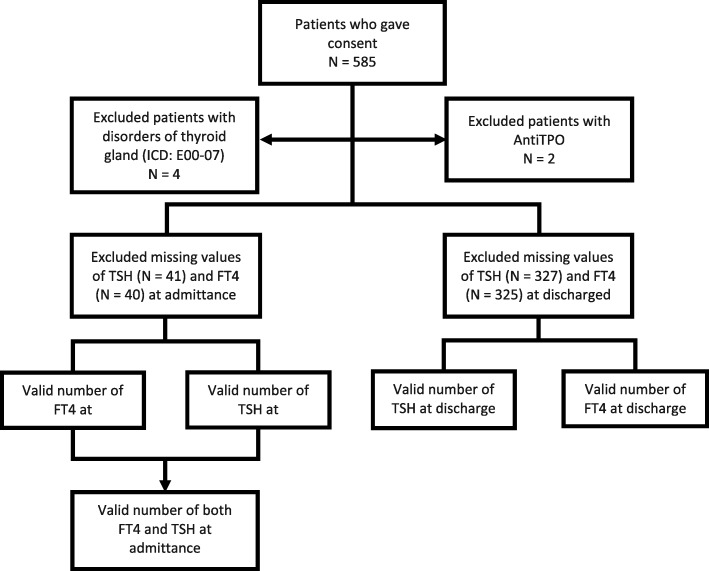
Inclusion and exclusion of patients

A total of 585 persons among the 1771 patients admitted in the study period gave written informed consent to participate. Some patients were excluded from these analyses because of disorders of the thyroid gland (International Classification of Diseases version 10 (ICD-10): E00–07) or because of the presence of anti-thyroid peroxidase antibodies (Anti-TPO). Also, patients for whom no measurements of FT4 or TSH were registered were excluded. As shown in Fig. 1, we included 539 patients with measurement for FT4 and 538 patients for TSH at admittance, and 254 and 252 patients at discharge, respectively. Data were available at both admittance and discharge for 239 patients for FT4 and for 236 patients for TSH.

Upon discharge, the patients’ psychiatric diagnoses were set according to ICD-10 research criteria. Final diagnoses were set in a consensus meeting with the physician or psychologist in charge of the patient’s treatment and at least two psychiatrists and/or senior clinical psychologist of whom at least one had personally examined the patient. In this study, the main diagnosis was used if multiple diagnoses were set. Diagnoses were categorized into 8 groups based on ICD-10 codes: Substance use disorder (SUD) (F10–19), schizophrenia spectrum disorder (F20–29), mania (F30–F31.2), bipolar depression (F31.3–31.5), unipolar depression (F32–F33), neurotic disorders (F40–49), and personality disorders (F60–69), and all other diagnoses were labeled as ‘other’.

Patient characteristics are shown in Table 1. Mean and median patient ages were 41 and 39 years, respectively. Mean and median days of duration of stay were 12 and 6, respectively. The patient distribution in diagnostic groups is shown in Table 2. After Bonferroni correction for multiple testing, still significantly more males than females were having SUD diagnoses (*x*^*2*^(*df*) = 19.528 (7), *p* = 0.007). For the other diagnostic groups, there was no difference in the gender distribution of diagnoses.

**Table 1:** Characteristics of patients at admittance

	FT4 (*N* = 539)	TSH (*N* = 538)
Gender		
Male	255	255
Female	284	283
Age		
years, mean ± SD	41.15 ± 16.93	41.09 ± 16.909
median	39.0	39.0
Duration of Stay		
days, mean ± SD	12.66 ± 35.320	12.69 ± 35.442
median	6.0	6.0

*FT4* free thyroxin, *TSH* thyroid-stimulating hormone

**Table 2:** Summary of the diagnostic groups distribution

Diagnostic Group	ICD	FT4 (N, Male/Female)	TSH (N, Male/Female)
SUD	F10–19	82 (51/31)^b^	83 (51/32)^b^
Schizophrenia	F20–29	92 (45/47)	91 (44/47)
Bipolar Mania	F30–31.2	32 (17/15)	32 (17/15)
Bipolar Depression	F31.3–31.5	18 (8/10)	18 (8/10)
Unipolar Depression	F32 - F33	115 (45/70)	114 (44/70)
Neurotic Disorders	F40–49	51 (17/34)	52 (18/34)
Personality Disorders	F60–69	33 (11/22)	34 (11/23)
Others	–	115 (61/54)	115 (62/53)
Statistics^a^		*x*^*2*^(*df*) = 19.528 (7), *p* = **0.007**	*x*^*2*^(*df*) = 18.613 (7), *p* = **0.009**

*SUD* substance use disorder, *ICD* International Classification of Diseases, *FT4* free thyroxin, *TSH* thyroid-stimulating hormone

^a^Chi-Square Test was conducted to examine a relationship between gender and diagnostic groups

^b^After Bonferroni correction, only SUD reaches significant

Entries in bold are significant at 0.05 level

### Blood samples and laboratory assays

As part of the standard clinical procedure, FT4 and TSH were measured in blood collected between 7:00 am and 10:00 am the first working day after admission. For some of the participants, the same analyses were done at discharge. Blood was analyzed the same day by immunochemiluminescent procedures at the clinical laboratory at St Olav’s University Hospital, Trondheim, Norway on Siemens ADVIA Centaur XPT. The reference ranges for TSH were between 0.56–4.89 mIU/L for persons 16–20 years old and 0.24–3.78 mIU/L for those above 20 years of age. The reference range for FT4 was 11.6–19.1 pmol/L.

### Statistics

Characteristics of patients at admittance, shown separately for TSH and FT4, were described as number of samples, age, gender, and duration of stay and were analyzed by descriptive analysis shown in frequencies, mean, standard deviation, and median. In this study, biological variables such as TSH and FT4 were used as continuous scales. The variable for diagnostic groups was used as categorical variable which contained 8 groups. A chi-Square test was conducted to examine the relationship between gender and diagnostic groups. Because the normality of continuous variables assessed by the Kolmogorov–Smirnov test were all skewed, non-parametric statistical analyses were chosen for further analysis. The levels of TSH and FT4 at admittance and at discharge were analyzed by descriptive statistics shown in frequencies, mean, median, and range, in addition to the Wilcoxon signed-ranks test which compares the levels of neuroendocrine biomarkers before and after hospitalization by using TSH or FT4 samples which are available at both admittance and discharge. As preliminary analysis, differences in the levels of TSH and FT4 across the diagnostic groups were assessed by a Kruskal-Wallis H-test both in total and for gender-separated samples. Lastly, the association between a specific psychiatric diagnostic group and the effect of TSH and FT4 in the model was assessed by binary logistic regression for psychiatric diagnostic groups including SUD, schizophrenia, bipolar mania, bipolar depression, unipolar depression, neurotic disorders, and personality disorders, both in total and for gender-separated samples.

If there was a statistically significant difference in any analysis, the Dunn-Bonferroni correction was used for *post-hoc* test to avoid multiple test problems. For statistical analysis, IBM-SPSS version 22 was used to assess all data. The results were considered significant when the *p* value was lower than 0.05.

## Results

### Deviation outside reference ranges of TSH and FT4 in total sample and in different genders and different diagnostic groups

1.7% of the participants had FT4 levels below reference range and 21.9% above. For TSH numbers were 2.6 and 5.8%, respectively. The deviation from the expected value of 2.5% both above and below each reference range is significant for both FT4 (*p* < 0.000) and TSH (*p* < 0.000) (calculations not shown). Comparing differences in distribution between diagnostic groups and thyroid hormone ranges did not reveal significant association for FT4 (*p* = 0.281), while there was a slightly significant difference regarding TSH (*p* = 0.047). There were no significant differences between genders regarding frequency of patients with levels of FT4 or TSH outside reference levels (Table 3).

**Table 3:** The relationship between FT4 and TSH levels in different ranges and gender as well as diagnostic groups

	FT4 Reference Range (≤ 11.6–19.1 ≤ (pmol/L))			TSH Reference Range (≤ 0.24–3.78 ≤ (mIU/L))		
	Below (n, %)	Within Reference Range (n, %)	Above (n, %)	Below (n, %)	Within Reference Range (n, %)	Above (n, %)
Total	9 (1.7%)	412 (76.4%)	118 (21.9%)	14 (2.6%)	493 (91.6%)	31 (5.8%)
Gender						
Male	6 (2.3%)	184 (72.2%)	65 (25.5%)	4 (1.6%)	240 (94.1%)	11 (4.3%)
Female	3 (1%)	228 (80.3%)	53 (18.7%)	10 (3.5%)	253 (89.4%)	20 (7.1%)
	*x*^*2*^(*df*) = 5.375 (2), *p* = 0.068			*x*^*2*^(*df*) = 4.081 (2), *p* = 0.13		
Diagnoses						
SUD	4 (5%)	64 (79%)	13 (16%)	4 (4.9%)	75 (91.5%)	3 (3.6%)
Schizophrenia	–	68 (73.9%)	24 (26.1%)	5 (5.5%)	84 (92.5%)	2 (2%)
Mania	–	22 (68.8%)	10 (31.2%)	–	30 (93.8%)	2 (6.2%)
Bipolar Depression	1 (2.9%)	26 (76.5%)	7 (20.6%)	1 (2.9%)	29 (85.3%)	4 (11.8%)
Unipolar Depression	–	90 (78.3%)	25 (21.7%)	1 (0.9%)	110 (96.5%)	3 (2.6%)
Neurotic Disorders	2 (3.9%)	41 (80.4%)	8 (15.7%)	1 (2%)	49 (94.2%)	2 (3.8%)
Personality Disorders	1 (3%)	25 (73.5%)	8 (23.5%)	1 (3%)	27 (81.8%)	5 (15.2%)
Others	1 (1%)	76 (76%)	23 (23%)	1 (1%)	89 (89%)	10 (10%)
	*x*^*2*^(*df*) = 16.543 (14), *p* = 0.281			*x*^*2*^(*df*) = 23.946 (14), *p* = **0.047**		

*SUD* substance use disorder, *TSH* thyroid-stimulating hormone, *FT4* free thyroxine

Chi-Square Test was conducted to examine a relationship between FT4 and TSH in above and below the reference ranges and gender as well as diagnostic groups

Reference range was set by 95% of healthy population

Entries in bold are significant at 0.05 level

### Differences in the levels of TSH or FT4 across psychiatric diagnosis in total and for gender-separated samples

Analyzing the total sample did not indicate any association between diagnoses and level of FT4 (*x*^*2*^(*df*) = 2.307(7), *p* = 0.941). For TSH however, an association was indicated (*x*^*2*^(*df*) = 14.062(7), *p* = 0.05, Additional file 1: Table S1). Separating the population into genders revealed significant differences in the levels of TSH between the diagnostic groups among females. SUD had the lowest mean score (mean score = 1.30 mIU/L), followed by schizophrenia (1.63 mIU/L), unipolar depression (1.70 mIU/L), neurotic disorders (1.85 mIU/L), bipolar mania (1.90 mIU/L), bipolar depression (2.10 mIU/L), other (2.27 mIU/L), and personality disorders (2.32 mIU/L) (Additional file 2: Table S2). A post hoc comparison by the Dunn-Bonferroni approach with adjusted *p*-value, confirmed significant difference among females between SUD and ‘other’, of which ‘other’ had significantly higher TSH levels (*Z* = − 3.202, *SE* = 18.321, *p* = 0.038) and this was confirmed in the regression model (Additional file 3: Table S3). No significant difference in the levels of TSH between different diagnostic groups was found for male patients.

### Variation in thyroid hormones from admittance to discharge

Data for FT4 were available at both admittance and discharge for 239 patients. Mean (median) levels of FT4 were 17.09 (16.75) pmol/L at admittance, and 16.07 (16.00) pmol/L at discharge. There was a statistically significant reduction from admittance to discharge (*n* = 239, *Z* = − 5.680, *p* = 0.00, Table 4).

**Table 4:** Descriptive statistics of FT4 and TSH (Admittance and Discharge)

	FT4 (pmol/L)		TSH (mIU/L)	
	Admittance	Discharge	Admittance	Discharge
N	538	252	539	254
Levels of Biomarkers				
mean	17.094	16.069	1.846	1.805
median	16.750	16.0	1.56	1.615
range	9.3–31.3	9.0–25.7	0.01–9.87	0.04–8.45
Statistics^a^	*Z* = −5.680, *p* = **0.00**		*Z* = −0.268, *p* = 0.789	

*FT4* free thyroxin, *TSH* thyroid-stimulating hormone

^a^Wilcoxon Signed-Ranks Test was conducted to compare difference between admittance and discharged

Entries in bold are significant at 0.05 level

Data for TSH were available at both admittance and discharge for 236 patients. Mean (median) levels of TSH at admittance were 1.85 (1.86) mIU/L, and 1.81 (1.62) mIU/L at discharged. As seen in Table 4, there was no significant difference in the levels of TSH at admittance and discharge (*n* = 236, *Z* = − 0.268, *p* = 0.789) (Table 4).

## Discussion

In a general psychiatric acute closed inpatient population with equal gender distribution, levels of FT4 and TSH at admittance deviated from levels expected in the general population with significantly more patients than expected having levels above reference range. There were no differences between neither genders nor different diagnostic groups regarding number of patients outside reference ranges for FT4 and TSH apart from a slight difference for TSH between diagnostic groups. Comparing absolute levels of TSH and FT4 revealed a significantly lower level of TSH in the group SUD compared to other diagnostic groups in females. No significant differences were seen between other diagnostic groups in females, and no significant difference between diagnostic groups was seen in males. Comparing levels of hormones at admittance and discharge (mean 12 / median 6 days later) showed a significant reduction in the level of FT4 during stay, whereas no significant change in the level of TSH was seen.

Hypo- and hyperthyroidism as a cause of symptoms mimicking depression and other psychiatric problems is a well-known clinical phenomenon. We find an increase in both FT4 and TSH over all psychiatric diagnostic groups (maybe except female SUD) without gender differences. Previous studies comparing specific diagnostic groups with healthy controls have found an increased FT4 in depression [5, 8, 14], bipolar disorders [3], and panic disorders [7]. Findings are however deviating [12]. Simultaneously, a decrease in FT4 has been described in patients with chronic alcohol use [15–18]. FT4 and TSH are linked. Associations between depression and level of TSH compared to healthy controls have been found in previous studies [8, 10]. In studies on cannabis users reduced levels of TSH have been reported [19, 20]. There was no significant association between FT4 and any of the diagnostic groups in our cohort. Only TSH showed a significant difference for one diagnostic group (SUD) and only in females. To our knowledge there are not many other studies on altered thyroid function in patients with substance use disorders except for alcohol and cannabis. As TSH was actually higher than expected in our cohort, the interpretation of our finding may simply be that women with SUD were the ones with most normal levels of TSH, all other diagnostic groups having increased levels. There is no obvious explanation for the absence of this effect in the male group. There are however gender differences regarding hormones [8] and this might contribute to altered sensitivity to stress as well as to psychoactive substances.

Psychotropic medication is of relevance in many psychiatric patients. Glucocorticoids, l-dopa and dopamine may reduce TSH-activity, while Li and dopamine-antagonists may increase it [21, 22]. Unfortunately, we did not register data on participants’ use of medications. The majority of patients would however not be on medications increasing thyroid function at admittance.

Our study comparing a broad variety of psychiatric diagnostic groups thus supplements the field indicating that the altered levels of thyroid hormones found in specific diagnostic groups compared to healthy controls is a broad psychiatric phenomenon reflecting psychic stress in general, not specific to certain diagnoses. This also is in line with the findings by Spratt in a general psychiatric acute inpatient population [23], although the potential variation over different diagnoses was not studied there. One explanation for the lack of relation between FT4 and diagnostic groups in our study could be the lack of power (small groups) or the acute-stress effect masking any differences between diagnostic groups, emphasizing the strength and potential clinical importance of the acute stress. This also emphasizes that altered thyroid function is not only potentially a cause of psychiatric symptoms but also, as postulated by Parry in 1825 [24], can be a result of emotional stress.

At admittance a higher number of patients than expected had FT4 above reference range. There then was a significant reduction in the level of FT4 from admittance to discharge. No significant change in TSH was seen. In a study with longer follow up (mean 21 days) in 22 acutely ill patients [23], a decrease in FT4 was seen, and in another longer follow up (mean 29 days) on acutely ill psychotic patients, both a decrease in FT4 and an increase in TSH were seen. However, despite a longer follow up than in our study, the increase in TSH was seen in fewer patients than those with a decrease in FT4 (41% vs 66%) [25]. Together, the findings indicate that the change in FT4 is not primarily driven by the central TSH as one could expect according to theories on the effect of psychiatric stress on the pituitary gland. Bunevicius suggests that the reduction in FT4 [25] is due to the use of antipsychotics. This would be in line with the mood stabilizer Li^+^ known to cause hypothyroidism by reducing production in the thyroid gland [26]. Bunevicius et al. however have no control group without medication preventing the disclosure of an effect independent of medications. Unfortunately we have not recorded use of medications in patients. However, as the whole diagnostic specter was represented no single group of medication would be used and not all patients would have medications. Besides, the significant number of patients with FT4 above reference range at admittance increases likelihood that a reduction for normalization is expected. The mechanism for this peripheral effect of stress on the thyroid gland could involve receptors for stress hormones such as cortisol and adrenaline in the thyroid gland; this, however, remains to be explored further.

Taken together, our findings do not link alterations in TSH / FT4 to any specific diagnoses but rather to psychiatric suffering in general. In line with this, there is a significant reduction in the level of FT4 over a mean 12-day (median 6-day) stay in a psychiatric acute ward.

### Limitations and strengths

There are several limitations to our study. The duration of stay was relatively short, lowering the possibility to detect a variation over time. However, at the same time, the registered variation in FT4 from admittance to discharge is thus more important because it reveals a dramatic change over a short time.

The number of participants is not large compared to epidemiological studies, although it is rather high for this kind of population and compared to other studies in acute-ward populations.

We do not have a group of healthy controls that could improve clarification of findings.

Data on patients’ use of medications is not included. As discussed above this is a weakness. However, our aim was to study the thyroid status in the psychiatric patients independent of the etiology of any deviation. This is interesting as deviating thyroid status may cause problems for the patients and need attention and treatment independently of its etiology.

All main psychiatric diagnostic groups were included in the study, and the wards represented the only closed, acute psychiatric inpatient service in the area, reducing the likelihood of social selection and loss of important groups. The possibility to use data for persons at the time they are acutely ill at admittance is a strength.

## Conclusion

Our study indicates that there is an overall increase in the level of FT4 and TSH in the acute psychiatric ill population independent of diagnoses and that there is a reduction (normalization) during the acute stay. There is no significant change in TSH, and the change in FT4 is quick (median duration of stay was 6 days), indicating that the change in FT4 is not driven by TSH. The effect of psychological stress via hypothalamus and TSH has been suggested previously; our study indicates that another mechanism must be causing this. Cortisol and adrenaline binding directly to the thyroid gland are candidates, although the mechanisms are, to our knowledge, not known.

The findings indicate that psychological stress may affect somatic functions in ways not known so far, a phenomenon that may have implications for treatment and somatic follow up of severely ill psychiatric patients.

## Additional files

Additional file 1: **Table S1.** Serum FT4 and TSH Levels across 8 Diagnostic Groups in Total Sample. (DOCX 19 kb)

Additional file 2: **Table S2**. Serum FT4 and TSH Levels across Different Diagnosis in Gender Separated Samples (Unadjusted Model). (DOCX 23 kb)

Additional file 3: **Table S3.** Associations between A Specific Diagnostic Group and the Set of Neuroendocrine Biomarkers in Total and Gender Separated Sample. (DOCX 30 kb)

## Abbreviations

Anti-TPO: Anti-thyroid peroxidase antibodies
FT4: Free thyroxine
HPT: Hypothalamic–Pituitary–Thyroid
ICD: International classification of diseases
SUD: Substance use disorder
T3: Triiodothyronine
T4: Thyroxine
TCA: Tricyclic antidepressants
TRH: Thyrotropin-releasing hormone
TSH: Thyroid Stimulating hormone
