# Supplementary material for: FT4 and TSH, relation to diagnoses in an unselected psychiatric acute-ward population, and change during acute psychiatric admission
Source: BMC Psychiatry. 2018 Jul 28;18:244. doi: 10.1186/s12888-018-1819-3 (PMC6064071; doi:10.1186/s12888-018-1819-3)
Supplement: Supplementary file 1 — Table S1. Serum FT4 and TSH Levels across 8 Diagnostic Groups in Total Sample. (DOCX 19 kb) [file 12888_2018_1819_MOESM1_ESM.docx]

| Table S1. Serum FT4 and TSH Levels across 8 Diagnostic Groups in Total Sample | | | | | | | | | | | |  |
| --- | --- | --- | --- | --- | --- | --- | --- | --- | --- | --- | --- | --- |
|  | SUD | Schizophrenia | Bipolar Mania | Bipolar Depression | Unipolar Depression | Neurotic Disorders | Personality Disorders | Others | Statistics* | | | |
|  | (F10 - 19) | (F20 - 29) | (F30 - 31.2) | (F31.3 - 31.5) | (F32 - F33) | (F40 - 49) | (F60 - 69) |  | *x^2^* | *df* | *p* | |
| FT4 (pmol/L) |  |  |  |  |  |  |  |  |  |  |  | |
| N | 82 | 92 | 32 | 18 | 115 | 51 | 34 (11 / 23) | 115 | 2.307 | 7 | 0.941 | |
| mean ± SD | 16.841 ± 3.189 | 17.475 ± 3.723 | 17.694 ± 3.358 | 16.756 ± 3.041 | 17.096 ± 2.856 | 16.70 ± 3.224 | 16.929 ± 3.171 | 17.077 ± 3.412 |  |  |  | |
| median | 16.9 | 16.8 | 17.4 | 16.0 | 16.6 | 16.8 | 15.8 | 16.8 |  |  |  | |
| range | 10.5 - 29.7 | 12.0 - 31.3 | 11.8 - 28.0 | 12.5 - 22.8 | 12.2 - 27.3 | 9.3 - 26.8 | 11.2 - 25.7 | 10.7 - 27.3 |  |  |  | |
| TSH (mIU/L) |  |  |  |  |  |  |  |  |  |  |  | |
| N | 83 | 91 | 32 | 18 | 114 | 52 | 33 | 115 | 14.062 | 7 | 0.050 | |
| mean ± SD | 1.599 ± 1.218 | 1.644 ± 1.128 | 1.953 ± 1.631 | 2.121 ± 1.287 | 1.823 ± 1.126 | 1.801 ± 1.126 | 2.12 ± 1.339 | 2.076 ± 1.30 |  |  |  | |
| median | 1.46 | 1.43 | 1.555 | 2.255 | 1.535 | 1.65 | 1.87 | 1.65 |  |  |  | |
| range | 0.10 - 9.18 | 0.02 - 7.55 | 0.40 - 9.87 | 0.17 - 5.57 | 0.10 - 7.6 | 0.02 - 5.99 | 0.17 - 5.27 | 0.01 - 6.68 |  |  |  | |
| SUD: Substance Use Disorder, FT4: Free Thyroxin, TSH: Thyroid-stimulating Hormone | | | | | | | | | | | |  |
| *The Kruskal Wallis test was used to compare median scores of TSH and FT4. | | | | | | | | | | | |  |
