# Supplementary material for: FT4 and TSH, relation to diagnoses in an unselected psychiatric acute-ward population, and change during acute psychiatric admission
Source: BMC Psychiatry. 2018 Jul 28;18:244. doi: 10.1186/s12888-018-1819-3 (PMC6064071; doi:10.1186/s12888-018-1819-3)
Supplement: Supplementary file 2 — Table S2. Serum FT4 and TSH Levels across Different Diagnosis in Gender Separated Samples (Unadjusted Model). (DOCX 23 kb) [file 12888_2018_1819_MOESM2_ESM.docx]

| Table S2. Serum FT4 and TSH Levels across Different Diagnosis in Gender Separated Samples (Unadjusted Model) | | | | | | | | | | | | |
| --- | --- | --- | --- | --- | --- | --- | --- | --- | --- | --- | --- | --- |
|  |  | SUD | Schizophrenia | Mania | Bipolar Depression | Unipolar Depression | Neurotic Disorders | Personality Disorders | Others | Statistics | | |
|  |  | (F10 - 19) | (F20 - 29) | (F30 - 31.2) | (F31.3 - 31.5) | (F32 - F33) | (F40 - 49) | (F60 - 69) |  | *x^2^* | *df* | *p* |
| FT4 (pmol/L) |  |  |  |  |  |  |  |  |  |  |  |  |
| *Male (N) |  | 51 | 45 | 17 | 8 | 45 | 17 | 11 | 61 |  |  |  |
| mean ± SD |  | 17.006 ± 3.223 | 17.842 ± 3.423 | 17.835 ± 2.724 | 16.088 ± 3.124 | 17.20 ± 2.504 | 17.094 ± 3.941 | 17.573 ± 4.021 | 17.777 ± 3.525 |  |  |  |
| **median |  | 17.0 | 17.0 | 17.4 | 15.4 | 16.7 | 18.0 | 17.2 | 17.4 | 3.916 | 7 | 0.789 |
| range |  | 10.5 - 29.7 | 12.6 - 28.8 | 13.3 - 23.1 | 12.5 - 20.6 | 12.2 - 22.4 | 9.3 - 24.8 | 11.2 - 25.7 | 11.9 - 27.3 |  |  |  |
| Female(N) |  | 31 | 47 | 15 | 10 | 70 | 34 | 23 | 54 |  |  |  |
| mean ± SD |  | 16.571 ± 3.164 | 17.123 ± 3.994 | 17.533 ± 4.054 | 17.290 ± 3.076 | 17.029 ± 3.076 | 16.503 ± 2.845 | 16.622 ± 2.725 | 16.285 ± 3.124 |  |  |  |
| **median |  | 16.8 | 16.0 | 16.70 | 16.6 | 16.45 | 16.55 | 15.4 | 15.95 | 2.473 | 7 | 0.929 |
| range |  | 11.5 - 25.5 | 12.0 - 31.3 | 11.8 - 28.0 | 12.9 - 22.8 | 12.8 - 27.3 | 11.9 - 26.8 | 12.5 - 22.6 | 10.7 - 25.4 |  |  |  |
| TSH (mIU/L) |  |  |  |  |  |  |  |  |  |  |  |  |
| *Male (N) |  | 51 | 44 | 17 | 8 | 44 | 18 | 11 | 62 |  |  |  |
| mean ± SD |  | 1.785 ± 1.455 | 1.656 ± 0.971 | 1.996 ± 2.098 | 2.153 ± 1.076 | 2.015 ± 1.374 | 1.706 ± 0.746 | 1.726 ± 0.999 | 1.909 ± 1.193 |  |  |  |
| **median |  | 1.49 | 1.44 | 1.48 | 2.205 | 1.78 | 1.695 | 1.47 | 1.545 | 4.255 | 7 | 0.750 |
| range |  | 0.16 - 9.18 | 0.18 - 3.79 | 0.40 - 9.87 | 0.87 - 4.18 | 0.41 - 7.60 | 0.62 - 3.51 | 0.60 - 3.57 | 0.01 - 6.68 |  |  |  |
| Female(N) |  | 32 | 47 | 15 | 10 | 70 | 34 | 22 | 53 |  |  |  |
| mean ± SD |  | 1.302 ± .60 | 1.632 ± 1.268 | 1.904 ± .920 | 2.095 ± 1.491 | 1.702 ± .966 | 1.851 ± 1.290 | 2.316 ± 1.461 | 2.270 ± 1.399 |  |  |  |
| **median |  | 1.35 | 1.39 | 1.70 | 2.255 | 1.485 | 1.635 | 2.295 | 1.91 | 16.234 | 7 | **0.023** |
| range |  | 0.10 - 2.19 | 0.02 - 7.55 | 0.72 - 4.19 | 0.17 - 5.57 | 0.10 - 4.76 | 0.02 - 5.99 | 0.17 - 5.27 | 0.25 - 6.67 |  |  |  |
| SUD: Substance Use Disorder, FT4: Free Thyroxine, TSH: Thyroid-stimulating Hormone | | | | | |  |  |  |  |  |  |  |
| *Chi-Square Test was conducted to examine a relationship between gender and diagnostic groups | | | | | |  |  |  |  |  |  |  |
| **The Kruskal Wallis test was used to compare median scores of TSH and FT4. | | | | | | | | | | | | |
