# Supplementary material for: FT4 and TSH, relation to diagnoses in an unselected psychiatric acute-ward population, and change during acute psychiatric admission
Source: BMC Psychiatry. 2018 Jul 28;18:244. doi: 10.1186/s12888-018-1819-3 (PMC6064071; doi:10.1186/s12888-018-1819-3)
Supplement: Supplementary file 3 — Table S3. Associations between A Specific Diagnostic Group and the Set of Neuroendocrine Biomarkers in Total and Gender Separated Sample. (DOCX 30 kb) [file 12888_2018_1819_MOESM3_ESM.docx]

| Supplementary Table 3. Associations between A Specific Diagnostic Group and the Set of Neuroendocrine Biomarkers in Total and Gender Separated Sample | | | | | | | | | | | | | | | | | | | | | | | |
| --- | --- | --- | --- | --- | --- | --- | --- | --- | --- | --- | --- | --- | --- | --- | --- | --- | --- | --- | --- | --- | --- | --- | --- |
|  |  | |  |  |  |  |  |  |  |  |  |  |  |  |  |  | |  |  |  |  |  |  |
|  |  | SUD | | |  | Schizophrenia | |  | Mania | |  | Bipolar Depression | |  | Unipolar Depression | | |  | Neurotic Disorders | |  | Personality Disorders | |
|  |  | *β* | | Odds Ratio |  | *β* | Odds Ratio |  | *β* | Odds Ratio |  | *β* | Odds Ratio |  | *β* | Odds Ratio | |  | *β* | Odds Ratio |  | *β* | Odds Ratio |
|  |  |  | |  |  |  |  |  |  |  |  |  |  |  |  |  | |  |  |  |  |  |  |
| Total (N = 534) |  |  | |  |  |  |  |  |  |  |  |  |  |  |  |  | |  |  |  |  |  |  |
| FT4 |  | -0.051 | | 0.950 |  | 0.031 | 1.032 |  | 0.065 | 1.068 |  | -0.020 | 0.980 |  | -0.006 | 0.994 | |  | -0.048 | 0.953 |  | 0.003 | 1.003 |
| TSH |  | -0.269 | | 0.764* |  | -0.16 | 0.852 |  | 0.104 | 1.110 |  | 0.142 | 1.153 |  | -0.031 | 0.969 | |  | -0.065 | 0.937 |  | 0.158 | 1.171 |
| Nagelkerke R^2^ |  | 0.019 | | |  | 0.012 | |  | 0.009 | |  | 0.006 | |  | 0.00 | | |  | 0.004 | |  | 0.008 | |
| Model Coefficients |  | *p* = **0**.**049** | | |  | *p* = 0.137 | |  | *p* = 0.435 | |  | *p* = 0.649 | |  | *p* = 0.937 | | |  | *p* = 0.578 | |  | *p* = 0.472 | |
|  |  |  | |  |  |  |  |  |  |  |  |  |  |  |  |  | |  |  |  |  |  |  |
| Male (n = 253) |  |  | |  |  |  |  |  |  |  |  |  |  |  |  |  | |  |  |  |  |  |  |
| FT4 |  | -0.060 | | 0.942 |  | .041 | 1.042 |  | 0.044 | 1.087 |  | -0.138 | 0.871 |  | -0.016 | 1.106 | |  | -0.047 | 0.954 |  | 0.007 | 1.007 |
| TSH |  | -0.085 | | 0.919 |  | -.153 | .858 |  | 0.096 | 1.126 |  | 0.105 | 1.111 |  | 0.101 | 0.985 | |  | -0.147 | 0.863 |  | -0.089 | 0.915 |
| Nagelkerke R^2^ |  | 0.01 | | |  | 0.014 | |  | 0.005 | |  | 0.028 | |  | 0.006 | | |  | 0.007 | |  | 0.002 | |
| Model Coefficients |  | *p* = 0.448 | | |  | *p* = 0.355 | |  | *p* = 0.764 | |  | *p* = 0.424 | |  | *p* = 0.647 | | |  | *p* = 0.726 | |  | *p* = 0.938 | |
|  |  |  | |  |  |  |  |  |  |  |  |  |  |  |  |  | |  |  |  |  |  |  |
| Female (n = 281) | |  | |  |  |  |  |  |  |  |  |  |  |  |  |  | |  |  |  |  |  |  |
| FT4 |  | -0.085 | | 0.919 |  | 0.021 | 1.021 |  | 0.083 | 1.087 |  | 0.075 | 1.078 |  | 0.003 | 1.006 | |  | -.032 | .969 |  | .031 | 1.031 |
| TSH |  | -0.706 | | 0.493** |  | -0.172 | 0.842 |  | 0.119 | 1.126 |  | 0.218 | 1.244 |  | -0.178 | 0.864 | |  | -.016 | .984 |  | .312 | 1.366 |
| Nagelkerke R^2^ |  | 0.075 | | |  | 0.012 | |  | 0.011 | |  | 0.013 | |  | 0.008 | | |  | 0.002 | |  | 0.029 | |
| Model Coefficients |  | *p* = **0.005** | | |  | *p* = 0.374 | |  | *p* = 0.577 | |  | *p* = 0.613 | |  | *p* = 0.45 | | |  | *p* = 0.871 | |  | *p* = 0.182 | |
| SUD: Substance Use Disorder, FT4: Free Thyroxin, TSH: Thyroid-stimulating Hormone  *: *p* < 0.05, **: *p* < 0.01  The Binominal Logistic Regression was used to estimate the association. | | | | | | | | | | | | | | | | |  | | | | | | |
